# Pattern and prognosis of distant metastases in nasopharyngeal carcinoma: A large‐population retrospective analysis

**DOI:** 10.1002/cam4.3301

**Published:** 2020-07-10

**Authors:** Weiling Qu, Sihan Li, Miao Zhang, Qiao Qiao

**Affiliations:** ^1^ Department of Radiation Oncology the First Hospital of China Medical University Shenyang, Liaoning China

**Keywords:** metastasis, nasopharyngeal carcinoma, nomogram, prognosis, SEER

## Abstract

Currently, the features and prognosis of nasopharyngeal carcinoma (NPC) with distant metastases are still rarely reported. Thus, the main purpose of our study was to investigate the metastasis patterns of different histological types of NPC and to clarify the prognostic characteristics of metastases at different sites. Patients were enrolled from the SEER program from 2010 to 2016. Chi‐squared tests were used to compare features between groups. The tendency to develop combined metastases was assessed with the odds ratio. The Kaplan‐Meier method was used for the survival analysis. Univariate and multivariate Cox analyses were used to select the independent prognostic risk factors for inclusion in the nomogram. In the present study, we found the following: (1) tumors are highly likely to metastasize if they have a larger volume, the regional lymph nodes are relatively large, or the regional lymph nodes are biopsied but not removed; (2) the bone and the brain were the most and least common metastatic sites among all histological types and N stages. Metastasis at two sites was the most common pattern, and bone metastasis was generally associated with metastasis to the liver or brain; (3) the prognostic analyses in metastatic patients showed that cancer‐specific survival (CSS) was relatively worse in patients with multiple metastases, and in those with liver metastasis regardless of the number of other metastatic sites; (4) A nomogram was constructed for clinical use based on four independent prognostic risk indicators, including histology, radiation therapy, chemotherapy, and metastatic status. Our findings provide a reference for clinical decision‐making and future diagnostic screening tests for NPC with distant metastases.

## Graphical abstract

Our study shows the distribution patterns and prognoses of distant metastases among 4 histological types in NPC. A nomogram was constructed for clinical use based on 4 independent prognostic risk indicators, including histology, radiation therapy, chemotherapy and metastatic status. Our findings provide a reference for clinical decision‐making and future diagnostic screening tests for NPC with distant metastases.

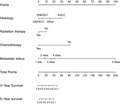

## 1 INTRODUCTION

Nasopharyngeal carcinoma is the most common malignant tumor of the head and neck in Southeast Asia and originates from the epithelium of the nasopharynx.^1^, ^2^ In a 2018 global report, morbidity due to nasopharyngeal carcinoma (NPC) accounted for approximately 0.7% of 36 cancers worldwide, with a total of 129 079 cases, and mortality due to NPC accounted for approximately 0.8% of all cancer‐related mortality, with a total of 72 987 deaths.^3^ Because of the widespread use of intensity‐modulated radiotherapy and chemotherapy in recent decades, the mortality of patients with NPC has decreased, and the 5‐year survival has generally improved.^1^, ^4^, ^5^ Currently, the main cause of death and the key obstacle to treatment are local recurrence and distant metastasis.^6^, ^7^, ^8^

The incidence of bone metastasis is approximately 64%‐67%, and bone is the most common site of metastases in NPC.^9^, ^10^, ^11^ In addition, because the nasopharynx is adjacent to a rich lymphatic network, poorly differentiated and undifferentiated NPCs are more likely to metastasize than other head and neck squamous cancers.^12^, ^13^ Compared with patients with regional metastases in only level II lymph nodes, patients with regional metastases in only level I, levels II‐IV, and levels II and IV lymph nodes have significantly higher risks of distant metastases.^14^ The median overall survival of patients with distant metastases at diagnosis was approximately 10‐36 months.^7^, ^15^, ^16^

Studies on this subject have been mostly restricted to limited comparisons of the relationship between macroscopic distant metastasis and survival. The specific effects of metastases to different organs and the number of distant metastases on prognosis need to be further investigated. Thus, new and comprehensive big data research is urgently needed to elucidate the detailed distribution characteristics and patterns of metastasis to guide clinical decision‐making and treatment of NPC patients with metastasis. This study was performed to explore the metastasis patterns of NPC and to identify the clinical and prognostic characteristics of different metastatic sites based on data from the Surveillance, Epidemiology, and End Results (SEER) database.

## 2 METHODS

We conducted a retrospective analysis of NPC patients using SEER*Stat software (version 8.3.6) using the data collected by the National Cancer Institute (NCI) SEER program since 1973. A detailed description of these data can be found on the official SEER website (https://seer.cancer.gov/data/).

### 2.1 Patient eligibility criteria

The inclusion criteria were as follows: (1) NPC with positive histological findings; (2) the first and only primary malignancy was NPC; (3) available follow‐up data; and (4) International Classification of Disease‐O‐3 (ICD‐O‐3) site codes C11.0‐C11.3, C11.8 and C11.9 and histologic codes 8010, 8020, 8021, 8070‐8073, 8082, and 8083. The exclusion criteria were as follows: (1) survival data were missing or unknown; (2) reporting source was autopsy or death certificate only; and (3) metastatic status was missing or unknown.

Based on the inclusion and exclusion criteria, a total of 2758 patients with metastatic information were enrolled in 2010‐2016. The metastasis data in the SEER database include metastases to the bone, brain, liver, lung, and distant lymph node (DL). According to the World Health Organization (WHO) classification scheme, the histological types of NPC were classified into keratinizing squamous cell carcinoma (KSCC; ICD‐O‐3 codes 8070, 8071), differentiated nonkeratinizing squamous cell carcinoma (DNKSCC; ICD‐O‐3 codes 8072, 8073), undifferentiated nonkeratinizing squamous cell carcinoma (UNKSCC; ICD‐O‐3 codes 8020, 8021, 8082, and 8083) and other unspecified groups (ICD‐O‐3 code 8010). All cases were classified according to the AJCC 7th edition staging system.

### 2.2 Statistical analysis

The cut‐off values for variables, including age at diagnosis, tumor size, and size of lymph nodes, were calculated by X‐tile software (version 3.6.1). The differences in baseline demographic characteristics between patients with and without metastatic disease were compared by Pearson's chi‐squared test or Fisher's exact test for categorical variables. Comparisons of metastatic distributions based on different pathological tissue types and N stage were performed using one‐way ANOVA. The odds ratios for each possible combination among the five different lesions were compared by Pearson's chi‐squared test or Fisher's exact test. Kaplan‐Meier curves were generated, and differences in survival were assessed with the log‐rank (Mantel‐Cox) test. Univariate and multivariate Cox regression analyses were performed to identify independent prognostic factors that were used to establish a nomogram (with the rms package in R). The predictive accuracy and discriminatory ability of the nomogram were assessed using the area under the curve (AUC) and calibration plot. All statistical tests were two‐tailed, and *P* < .05 was considered statistically significant. Statistical analyses were conducted using IBM SPSS Statistics software version 23, R version 3.6.1 and GraphPad Prism version 8.

## 3 RESULTS

### 3.1 Patient characteristics

In total, 2758 patients were included in our study, of whom 332 had distant metastases. A total of 909 of 2758 tumors (33.0%) were KSCC, 820 tumors (29.7%) were DNKSCC, 515 tumors (18.7%) were UNKSCC, and 514 tumors (18.6%) did not belong to any specified groups (other group).

All of the baseline characteristics of the patients are summarized in Table 1 and Table S1. The two groups had significant differences in histology, tumor size, and size of lymph nodes. With regard to treatment strategies, the metastatic group underwent less radiation therapy and more regional lymph node biopsies without excision than the nonmetastatic group.

**TABLE 1:** Baseline clinical characteristics in NPC

Characteristics	No metastasis		Metastasis		*P* value
	Number	%	Number	%	
Age at diagnosis					
≤50	932	38.4	122	36.7	0.085
50‐70	1210	49.9	157	47.3	
>70	284	11.7	53	16.0	
Gender					
Male	1705	70.3	263	79.2	0.001
Female	721	29.7	69	20.8	
Marital status					
Married	1412	58.2	172	51.8	0.079
Unmarried	868	35.8	135	40.7	
Unknown	146	6.0	25	7.5	
Race recode					
White	1049	43.2	126	38.0	0.240
Black	290	12.0	48	14.5	
Other_†_	1059	43.7	155	46.7	
Unknown	28	1.2	3	0.9	
Grade					
I	35	1.4	4	1.2	0.469
II	187	7.7	23	6.9	
III	729	30.0	117	35.2	
IV	738	30.4	93	28.0	
Unknown	737	30.4	95	28.6	
Histology					
KSCC	803	33.1	106	31.9	0.013
DNKSCC	738	30.4	82	24.7	
UNKSCC	453	18.7	62	18.7	
Other	432	17.8	82	24.7	
T stage					
T0_‡_	11	0.5	2	0.6	<0.0001
T1	784	32.3	71	21.4	
T2	441	18.2	44	13.3	
T3	480	19.8	65	19.6	
T4	610	25.1	100	30.1	
TX	91	3.8	50	15.1	
Unknown	9	0.4	0	0	
N stage					
N0	526	21.7	40	12.0	<0.0001
N1	805	33.2	84	25.3	
N2	732	30.2	105	31.6	
N3	313	12.9	92	27.7	
NX	41	1.7	11	3.3	
Unknown	9	0.4	0	0	
Scope Reg LN Sur_§_					
None	1864	76.8	239	72.0	0.010
Reg LN biopsy	356	14.7	61	18.4	
Reg LN removed	199	8.2	27	8.1	
Unknown	7	0.3	5	1.5	
Radiation therapy					
Yes	2168	89.4	194	58.4	<0.0001
No	258	10.6	138	41.6	
Chemotherapy					
Yes	2017	83.1	273	82.2	0.678
No	409	16.9	59	17.8	
Tumor size(mm)					
Microscopic focus_¶_	1	0	0	0	0.001
≤30	515	21.2	50	15.1	
30‐60	844	34.8	95	28.6	
>60	169	7.0	30	9.0	
Unknown	897	37.0	157	47.3	
Size of lymph nodes(mm)					
No involved regional lymph nodes	498	20.5	34	10.2	<0.0001
Microscopic focus_¶_	1	0	0	0	
≤10	93	3.8	7	2.1	
10‐55	1124	46.3	151	45.5	
>55	153	6.3	28	8.4	
Unknown	557	23.0	112	33.7	

Abbreviations: Microscopic focus_¶_, Microscopic focus or foci only, no size of focus is given; Other_†_, American Indian, Alaska Native, Asian, Pacific Islander; Scope Reg LN Sur_§_, Scope regional lymph nodes surgery; T0_‡_, Epstein‐Barr virus (EBV) positive‐unknown primary cancer with cervical lymph node involvement.

Furthermore, we investigated the prognostic indicators for cancer‐specific survival (CSS) in NPC patients. A total of 20 variables were extracted and assessed with univariate Cox analysis, and 15 variables with statistically significant differences were included in multivariate Cox regression models. Eventually, we determined the eight independent prognostic factors for CSS in NPC patients, including metastatic status (*P* = .002). Only liver metastasis (HR 9.169, 95% CI 3.154‐26.653), dual‐site metastasis (HR 2.779, 95% CI 1.500‐5.148), and tetra‐site metastasis (HR 9.520, 95% CI 1.168‐77.564) were all related to poor CSS. Furthermore, metastatic status was also identified as one of the independent risk factors for overall survival (OS) in the univariate and multivariate Cox analyses (*P* = .027, Tables S2 and S3).

### 3.2 Metastatic patterns

**FIGURE 1:**
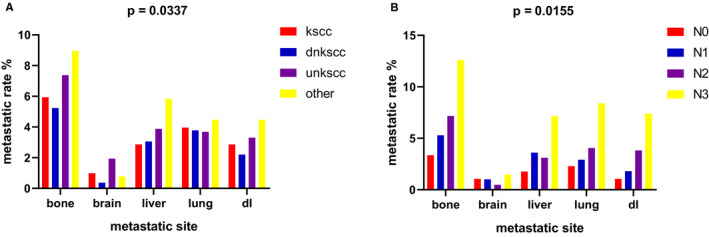
Distribution of distant metastatic organs among four histological types and 4 N stages

As mentioned above, 332 of 2758 patients (12.0%) had distant metastases, and 325 of 332 patients (97.9%) had metastases in five organs (bone, brain, liver, lung, and DL). According to histological subtype, NPC was divided into KSCC, DNKSCC, UNKSCC, and other group for the comparison of the metastatic distribution. Figure 1A clearly shows that bone was the most common metastatic organ and brain was the least common metastatic organ among all histological subtypes of NPC. It is worth noting that there were some differences in the metastatic patterns among different subtypes. Without considering the other group, UNKSCC was the most common histological subtype with bone, brain, liver, and DL metastases, while KSCC was the most common histological subtype with lung metastases. On the other hand, DNKSCC was the least common histological type with bone, brain, and DL metastases. KSCC and UNKSCC were the least common histological types with liver metastasis and lung metastasis respectively. Similarly, Figure 1B shows that bone was the most frequent metastatic site among all N stages, and the brain was the least common metastatic site. Moreover, the N3 stage had the highest incidence of metastases in any of the five investigated organs.

### 3.3 Combination of metastases

**FIGURE 2:**
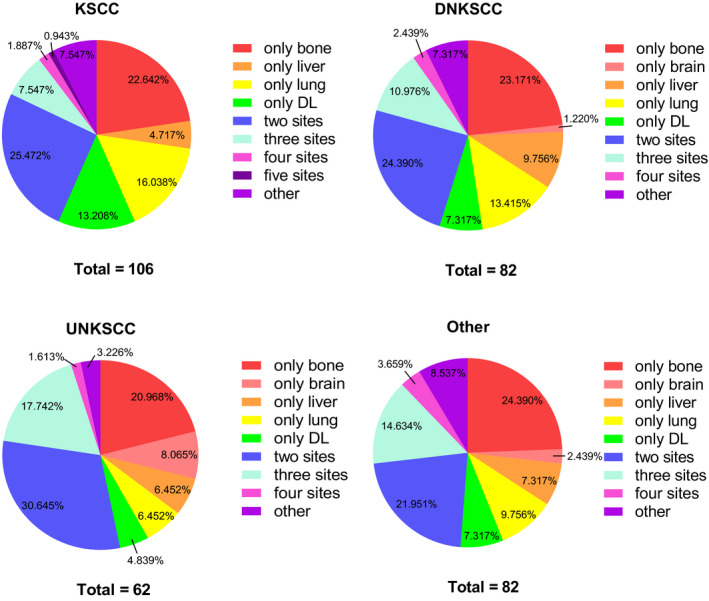
Percentages of single and multiple metastases among different histological types

Of the 332 tumors with metastases, 106 were KSCC, 82 were DNKSCC, 62 were UNKSCC, and the remaining 82 belonged to the other group. Some patients had only one metastasis, and others had multiple metastases that occurred simultaneously or sequentially. We generated pie charts to illustrate the proportions of each single metastasis and combined metastases in each subtype of NPC. Based on Figure 2, we concluded the following rules: (1) of the single metastases, solitary bone metastasis was the most common among all histological types except the other group. Solitary lung metastasis was the second most common metastasis in KSCC and DNKSCC, and brain metastasis alone was the second most common metastasis in UNKSCC. (2) The incidence of dual‐site metastasis was higher than that of single metastasis in all subtypes. (3) The frequency of different metastasis patterns among each histological subtype was as follows: two sites > three sites > four sites > five sites.

**FIGURE 3:**
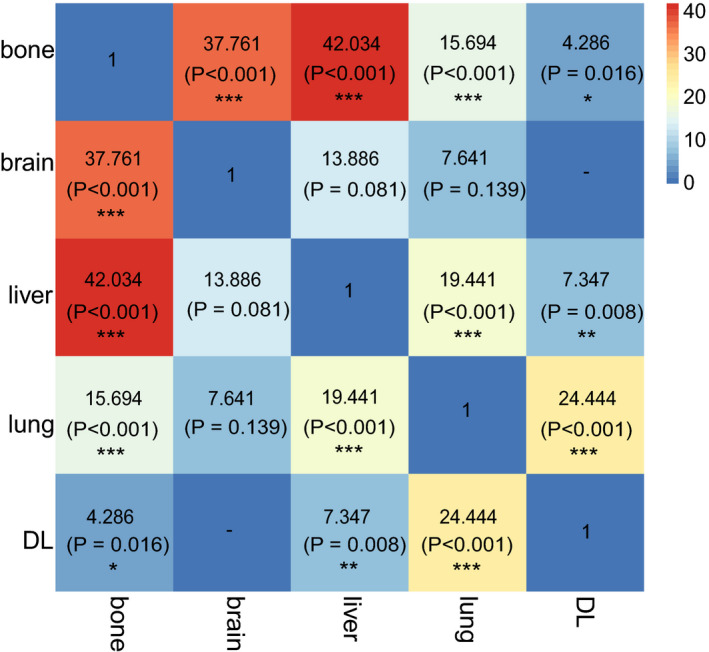
Odds ratios comparison of different dual‐site metastasis combinations (*P < .05, **P < .01, ***P < .001)

To better explain the metastatic interactions among cases of dual‐site metastasis, we compared the odds ratios of each possible combination for all five organs. Bone metastasis was more likely to co‐occur with liver metastasis (OR: 42.034) or brain metastasis (OR: 37.761) than with other lesions, and lung metastasis was more likely to co‐occur with liver metastasis (OR: 19.441) or DL metastasis (OR: 24.444) than with other metastases (Figure 3).

### 3.4 Prognostic analysis

In our study, 812 of 2758 (29.4%) patients died, and 206 of 332 (62.0%) patients with metastatic diseases died. Metastatic disease was confirmed as an independent prognostic factor for mortality in patients with NPC, and patients with metastases had a worse CSS than those without metastases (Figure S1).

Moreover, we explored the prognostic markers in 332 NPC patients with metastases. The 18 variables shown in Table 2 were subjected to univariate Cox analysis, and 6 variables with statistically significant differences were selected for analysis with multivariate Cox regression. Ultimately, we identified four independent prognostic factors affecting CSS in patients with metastatic disease (Table 3): histology, radiation therapy, chemotherapy, and metastatic status. Patients with metastatic disease who underwent radiation therapy achieved a better CSS (HR 0.725, 95% CI 0.529‐0.994) and OS (HR 0.689, 95% CI 0.509‐0.933) than those who did not undergo radiation therapy. Patients with metastatic disease who underwent chemotherapy also achieved a better CSS (HR 0.328, 95% CI 0.222‐0.483) and OS (HR 0.334, 95% CI 0.230‐0.484) than those who did not undergo chemotherapy. Patients with metastatic disease with KSCC had worse CSS and OS than patients with the other three histological subtypes of NPC (*P* < .001). Compared to patients with single‐site metastasis, those with tri‐site and penta‐site metastases had significantly worse CSS and OS; however, the p values for dual‐site and tetra‐site metastases did not reach significance (Table 3).

**TABLE 2:** Univariate Cox variance analysis of CSS and OS in NPC with metastasis

Variables	CCS		OS	
	HR (95% CI_†_)	*P* value	HR (95% CI_†_)	*P* value
Age at diagnosis		0.025		0.003
≤50	Reference		Reference	
50‐70	1.250 (0.913‐1.711)	0.164	1.243 (0.917‐1.686)	0.161
>70	1.811 (1.178‐2.783)	0.007	2.009 (1.341‐3.011)	0.001
Gender		0.026		0.018
Male	Reference		Reference	
Female	0.659 (0.456‐0.951)	0.026	0.653 (0.459‐0.930)	0.018
Marital status		0.716		0.942
Married	Reference		Reference	
Unmarried	0.946 (0.703‐1.274)	0.716	1.011 (0.759‐1.346)	0.942
Race recode		0.392		0.628
White	Reference		Reference	
Black	0.795 (0.511‐1.238)	0.310	0.864 (0.566‐1.320)	0.500
Other_‡_	0.826 (0.607‐1.124)	0.225	0.874 (0.649‐1.177)	0.374
Grade		0.191		0.391
I	Reference		Reference	
II	0.553 (0.125‐2.439)	0.434	0.635 (0.145‐2.773)	0.546
III	0.624 (0.152‐2.559)	0.513	0.642 (0.157‐2.628)	0.537
IV	0.429 (0.104‐1.778)	0.244	0.490 (0.119‐2.022)	0.324
Histology		<0.001		<0.001
KSCC	Reference		Reference	
DNKSCC	0.575 (0.395‐0.837)	0.004	0.584 (0.406‐0.841)	0.004
UNKSCC	0.417 (0.272‐0.639)	<0.001	0.459 (0.307‐0.685)	<0.001
Other	0.630 (0.432‐0.917)	0.016	0.611 (0.425‐0.880)	0.008
T stage		0.700		0.377
T0	Reference		Reference	
T1	0.515 (0.070‐3.768)	0.513	0.277 (0.067‐1.152)	0.078
T2	0.546 (0.073‐4.060)	0.554	0.291 (0.069‐1.232)	0.094
T3	0.631 (0.086‐4.615)	0.650	0.334 (0.080‐1.389)	0.132
T4	0.632 (0.087‐4.584)	0.650	0.332 (0.081‐1.364)	0.126
TX	0.745 (0.101‐5.477)	0.773	0.399 (0.095‐1.669)	0.208
N stage		0.606		0.231
N0	Reference		Reference	
N1	1.144 (0.703‐1.861)	0.588	1.123 (0.705‐1.788)	0.626
N2	0.895 (0.553‐1.448)	0.651	0.849 (0.533‐1.352)	0.491
N3	1.047 (0.649‐1.691)	0.850	0.998 (0.629‐1.584)	0.993
NX	1.571 (0.641‐3.850)	0.323	1.919 (0.867‐4.245)	0.108
Scope Reg LN Sur_§_		0.948		0.889
None	Reference		Reference	
Reg LN biopsy	1.012 (0.699‐1.464)	0.949	0.991 (0.692‐1.418)	0.960
Reg LN removed	0.924 (0.558‐1.531)	0.759	0.886 (0.543‐1.446)	0.628
Radiation therapy		0.001		<0.001
No	Reference		Reference	
Yes	0.616 (0.463‐0.821)	0.001	0.608 (0.462‐0.802)	<0.001
Chemotherapy		<0.001		<0.001
No	Reference		Reference	
Yes	0.301 (0.214‐0.424)	<0.001	0.296 (0.213‐0.410)	<0.001
Metastatic status		0.004		0.012
Single metastasis	Reference		Reference	
Dual‐site metastasis	1.159 (0.824‐1.629)	0.397	1.126 (0.811‐1.564)	0.478
Tri‐site metastasis	1.420 (0.917‐2.199)	0.116	1.356 (0.891‐2.064)	0.155
Tetra‐site metastasis	1.651 (0.721‐3.779)	0.236	1.524 (0.668‐3.480)	0.317
Penta‐site metastasis	42.221 (5.254‐339.276)	<0.001	30.640 (3.938‐238.406)	0.001
Tumor size(mm)		0.756		0.657
≤30	Reference		Reference	
30‐60	0.921 (0.565‐1.502)	0.741	0.822 (0.522‐1.293)	0.396
>60	1.126 (0.616‐2.056)	0.700	0.963 (0.539‐1.718)	0.897
Size of Lymph Nodes(mm)		0.838		0.637
No involved regional lymph nodes	Reference		Reference	
≤10	0.901 (0.268‐3.021)	0.865	1.135 (0.391‐3.296)	0.815
10‐55	0.825 (0.514‐1.323)	0.424	0.799 (0.503‐1.269)	0.342
>55	0.960 (0.523‐1.761)	0.896	0.990 (0.551‐1.778)	0.973
Level I‐III lymph nodes	1.028 (0.971‐1.088)	0.347	1.026 (0.971‐1.084)	0.359
Level IV‐V and retropharyngeal lymph nodes	1.038 (0.973‐1.108)	0.254	1.034 (0.971‐1.102)	0.300
Level VI‐VII and facial lymph nodes	0.909 (0.758‐1.091)	0.306	0.901 (0.750‐1.082)	0.264
Parapharyngeal, parotid, and suboccipital/retroauricular lymph nodes	0.866 (0.677‐1.109)	0.255	0.860 (0.674‐1.097)	0.226

Abbreviations: CI_†_, confidence interval; Other_‡_, American Indian, Alaska Native, Asian, Pacific Islander; Scope Reg LN Sur_§_, Scope regional lymph nodes surgery.

**TABLE 3:** Multivariate Cox variance analysis of CSS and OS in NPC with metastasis

Variables	CCS		OS	
	HR (95% CI_†_)	P value	HR (95% CI_†_)	P value
Age at diagnosis		0.457		0.171
≤50	Reference		Reference	
50‐70	1.034 (0.738‐1.448)	0.846	1.046 (0.754‐1.450)	0.788
>70	1.359 (0.823‐2.242)	0.231	1.537 (0.959‐2.465)	0.074
Gender		0.072		0.059
Male	Reference		Reference	
Female	0.696 (0.469‐1.033)	0.072	0.695 (0.477‐1.015)	0.059
Histology		<0.001		<0.001
KSCC	Reference		Reference	
DNKSCC	0.470 (0.311‐0.710)	<0.001	0.490 (0.329‐0.730)	<0.001
UNKSCC	0.419 (0.267‐0.659)	<0.001	0.466 (0.305‐0.713)	<0.001
Other	0.659 (0.441‐0.983)	0.041	0.659 (0.448‐0.970)	0.035
Radiation therapy		0.046		0.016
No	Reference		Reference	
Yes	0.725 (0.529‐0.994)	0.046	0.689 (0.509‐0.933)	0.016
Chemotherapy		<0.001		<0.001
No	Reference		Reference	
Yes	0.328 (0.222‐0.483)	<0.001	0.334 (0.230‐0.484)	<0.001
Metastatic status		0.026		0.049
Single metastasis	Reference		Reference	
Dual‐site metastasis	1.249 (0.869‐1.795)	0.229	1.212 (0.854‐1.719)	0.282
Tri‐site metastasis	1.688 (1.059‐2.689)	0.028	1.628 (1.040‐2.550)	0.033
Tetra‐site metastasis	2.020 (0.866‐4.712)	0.104	1.867 (0.804‐4.335)	0.146
Penta‐site metastasis	12.344 (1.469‐103.728)	0.021	9.319 (1.148‐75.675)	0.037

Note

Abbreviation: CI_†_, confidence interval.

**FIGURE 4:**
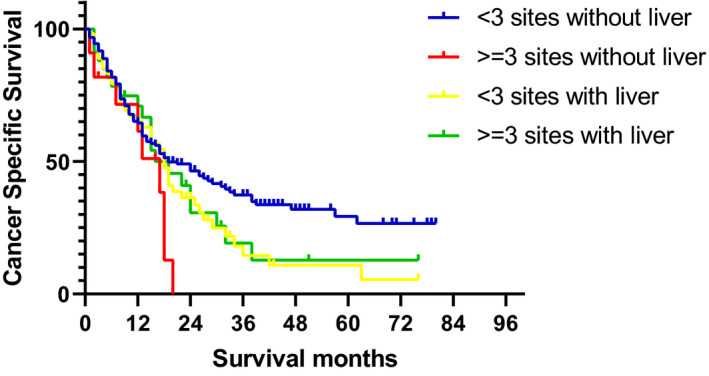
Intergroup survival analyses based on the number of metastatic sites and the existence of liver metastasis

Additionally, in patients with metastasis to a single organ, patients with bone metastasis (HR 2.227, 95% CI 1.118‐4.434), liver metastasis (HR 3.142, 95% CI 1.350‐7.311), and lung metastasis (HR 2.870, 95% CI 1.386‐5.942) had worse CSS than those with DL metastasis. Similar results were observed for OS (Table S4). However, there was no significant difference in CSS and OS between the patients with only brain metastasis and patients with only DL metastasis. In patients with extrahepatic metastasis, those with more than two metastatic sites had a worse CSS than those with one or two metastatic sites (*P* = .0377). However, a difference in survival time based on the number of metastatic sites was not observed in patients with liver metastasis (*P* = .7931, Figure 4).

**FIGURE 5:**
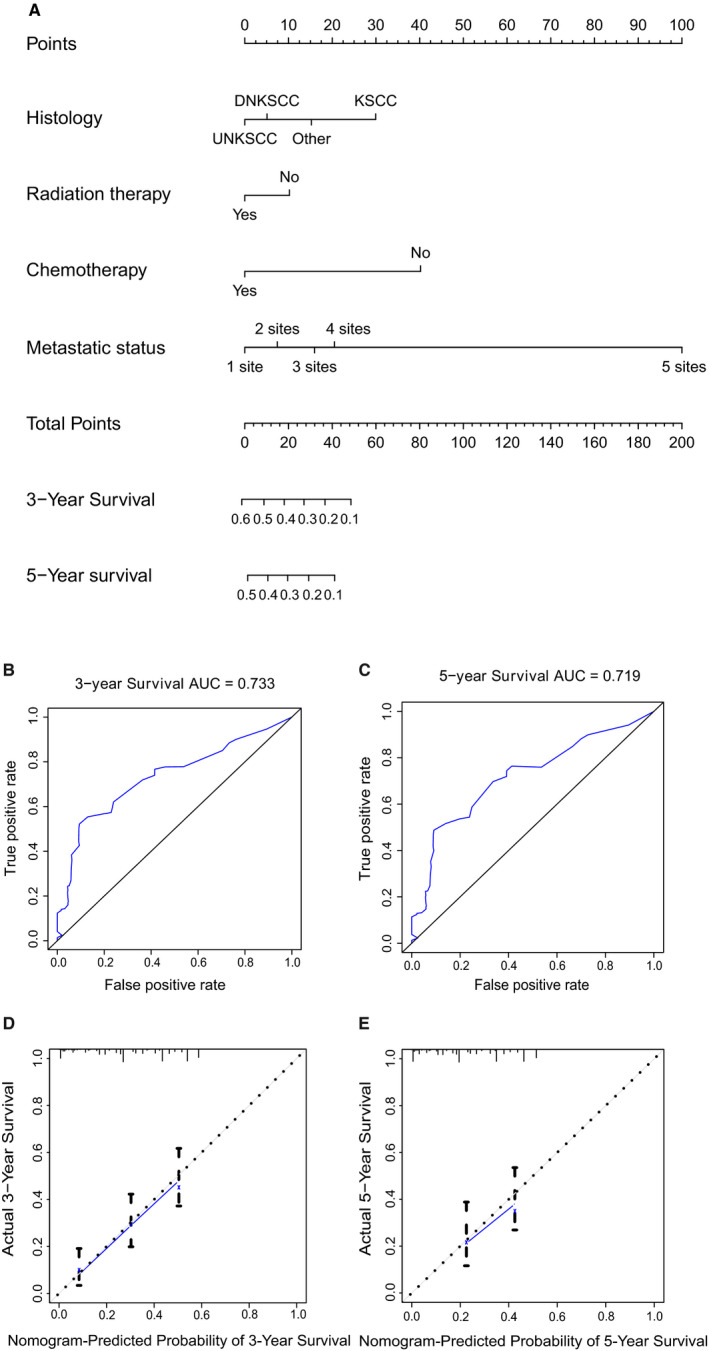
(A) Nomogram model for the prediction of 3‐year and 5‐year CSS in metastatic NPC patients. (B, C) AUC of the model for the prediction of 3‐year and 5‐year CSS in metastatic NPC patients. (D, E) Calibration curves of the comparison between the nomogram‐predicted and actual CSS at 3 and 5 years

Then, we constructed a nomogram that included the four independent risk indicators (Figure 5A). The AUC for the prediction of 3‐year CSS was 0.733 and that for the prediction of 5‐year CSS was 0.719. The calibration curves showed that the nomogram predictions were well‐correlated with the actual observations for 3‐year and 5‐year CSS (Figure 5B‐E).

## 4 DISCUSSION

As mentioned in the literature review, NPC patients with distant metastases generally had worse CSS and OS than those without metastases. Thus, it is very important to understand distant metastasis patterns. To the best of our knowledge, this is the first SEER‐based report of the patterns and prognosis of patients with NPC and distant metastasis to consider the influence of histological type.

Prior studies have shown that bone has the highest incidence of metastasis in NPC, and the brain has the lowest incidence.^17^, ^18^, ^19^ It is also well‐known that the histological type is an independent prognostic risk factor.^20^ Previous studies have noted that N3 stage is an independent predictor of poor prognosis in patients with NPC,^21^, ^22^, ^23^ which was consistent with the results of our study. We further found that in any of the five investigated organs, distant metastases were more common in patients with stage N3 disease than in those with other N stages, which may be related to extranodal extension (ENE) of the N3 stage.^24^

Very few studies have been performed on the patterns and tendencies of combined metastases in NPC. Our report showed that the incidence of dual‐site metastasis was higher than that of single‐site metastasis, which may be related to the change in the tumor microenvironment after metastasis to one organ.^25^, ^26^ Regarding the tendency to develop metastases in two organs, bone metastasis was more likely to be combined with liver or brain metastasis, and lung metastasis was more likely to be combined with liver or DL metastasis. Although the sequence of combined organ metastases was not available in the SEER database, understanding the trends in metastatic combinations would be important for risk assessment and diagnostic screening in NPC patients with advanced‐stage disease.

According to the baseline clinicopathological characteristics, the occurrence of distant metastasis is related to tumor size, lymph node involvement, and lymph node size. Distant metastasis is more common in patients with biopsied but not removed lymph nodes and is most common in patients with stage T4 or N3 tumors. Considering the possible impact of metastases on the CSS of NPC patients, multivariate Cox models were built, and metastatic status was identified as an independent prognostic indicator. Further univariate and multivariate Cox analyses were performed for 332 metastatic patients, and four independent prognostic markers were determined. More metastatic lesions were associated with worse CSS. Interestingly, the prognosis of patients with liver metastasis did not change based on the number of metastatic sites. In accordance with our results, previous studies have demonstrated that liver metastasis is associated with a dramatically worse prognosis regardless of the number of metastatic sites.^10^

There are still several potential limitations of our study that need to be mentioned. First, this was a retrospective study, and we only selected patients from 2010 to 2016 because the SEER database only started collecting metastasis information for NPC in 2010. Second, these patients had already had metastases at the initial diagnosis, and patients who developed metastases during the treatment process were not included. Additionally, the specific numbers, sizes, and orders of the metastases in these five organs were not available. Third, the plasma level of Epstein‐Barr virus (EBV) DNA is a sensitive prognostic tool for monitoring disease status in NPC but was not included in the SEER database.^27^, ^28^, ^29^ Consequently, based on the patterns of metastases to one or two sites, the EBV DNA levels and the first metastatic site are also recommended for inclusion in predictive models used to support prognostic assessments and treatment decisions. Furthermore, the survival curves in Figure 4 overlapped before 18 months, which limited the value of this result. Nevertheless, we are relatively more concerned about the long‐term survival of patients with distant metastases.

## 5 CONCLUSIONS

Our study shows the distribution patterns and prognoses of distant metastases in NPC. Interestingly, tumors with larger volumes and larger regional lymph nodes, as well as tumors with regional lymph nodes biopsied but not completely resected, had an increased probability of metastasis. Bone and brain were the most common and least common metastatic sites. Dual‐site metastasis was the most common pattern, and bone metastasis was most likely to co‐occur with liver or brain metastasis. Moreover, we identified four independent prognostic indicators that were incorporated into the nomogram. Metastases to multiple sites were associated with relatively worse survival. However, in patients with liver metastases, no difference in survival was observed between patients with different numbers of metastatic sites. Our findings provide more insights into clinical decision‐making and future diagnostic screening tests.

## CONFLICTS OF INTEREST

None declared.

## AUTHOR CONTRIBUTIONS

W Qu and Q Qiao have been involved in all aspects of design, interpretation, and writing; W Qu, S Li, and M Zhang have made substantial contributions to acquisition of data, or analysis and interpretation of data. All authors have given final approval of the version to be published. All authors agree to be accountable for all aspects of the work in ensuring that questions related to the accuracy or integrity of any part of the work are appropriately investigated and resolved.

## DATA AVAILABILITY STATEMENT

All data included in this study are available upon request from the corresponding author.

## Supporting information

Figure S1

Table S1

Table S2‐S3

Table S4
