# Supplementary figures and images for: Pattern and prognosis of distant metastases in nasopharyngeal carcinoma: A large‐population retrospective analysis
Source: Cancer Med. 2020 Jul 10;9(17):6147–58. doi: 10.1002/cam4.3301 (PMC7476823; doi:10.1002/cam4.3301)

## metastasis

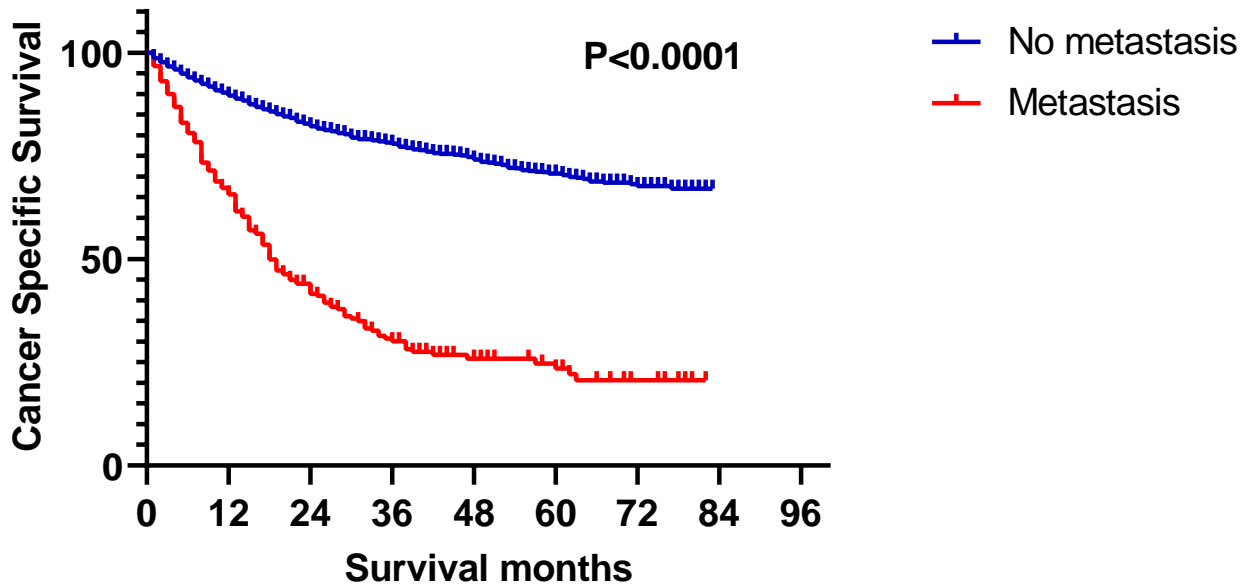

Supplement: Supplementary file 1 — Figure S1 [file CAM4-9-6147-s001.pdf]
