# Supplementary material for: Pattern and prognosis of distant metastases in nasopharyngeal carcinoma: A large‐population retrospective analysis
Source: Cancer Med. 2020 Jul 10;9(17):6147–58. doi: 10.1002/cam4.3301 (PMC7476823; doi:10.1002/cam4.3301)
Supplement: Supplementary file 2 — Table S1 [file CAM4-9-6147-s002.docx]

**Supplementary table 1: Baseline clinical characteristics in NPC**

| **Characteristics** | **No metastasis** | | **Metastasis** | | **P value** |
| --- | --- | --- | --- | --- | --- |
|  | **Number** | **%** | **Number** | **%** |  |
| **Level I-III lymph nodes** |  |  |  |  | **<0.0001** |
| No involvement in levels I, II or III lymph nodes | 781 | 32.2 | 68 | 20.5 |  |
| Level I lymph node(s) involved | 85 | 3.5 | 12 | 3.6 |  |
| Level II lymph node(s) involved | 711 | 29.3 | 81 | 24.4 |  |
| Level III lymph node(s) involved | 47 | 1.9 | 4 | 1.2 |  |
| Levels I and II lymph node(s) involved | 99 | 4.1 | 16 | 4.8 |  |
| Level I and III lymph node(s) involved | 11 | 0.5 | 1 | 0.3 |  |
| Level II and III lymph node(s) involved | 392 | 16.2 | 59 | 17.8 |  |
| Level I, II and III lymph node(s) involved | 101 | 4.2 | 34 | 10.2 |  |
| Unknown | 199 | 8.2 | 57 | 17.2 |  |
| **Level IV-V and retropharyngeal lymph nodes** |  |  |  |  | **<0.0001** |
| No involvement in levels IV or V or retropharyngeal lymph nodes | 1480 | 61.0 | 144 | 43.4 |  |
| Level IV lymph node(s) involved | 116 | 4.8 | 19 | 5.7 |  |
| Level V lymph node(s) involved | 245 | 10.1 | 35 | 10.5 |  |
| Retropharyngeal lymph node(s) involved | 175 | 7.2 | 21 | 6.3 |  |
| Level IV and V lymph node(s) involved | 91 | 3.8 | 33 | 9.9 |  |
| Level IV and retropharyngeal lymph node(s) involved | 12 | 0.5 | 1 | 0.3 |  |
| Level V and retropharyngeal lymph node(s) involved | 55 | 2.3 | 6 | 1.8 |  |
| Level IV, V and retropharyngeal lymph node(s) involved | 39 | 1.6 | 14 | 4.2 |  |
| Unknown | 213 | 8.8 | 59 | 17.8 |  |

**Supplementary table 1: Baseline clinical characteristics in NPC (continued)**

| **Level VI-VII and facial lymph nodes** |  |  |  |  | **<0.0001** |
| --- | --- | --- | --- | --- | --- |
| No involvement in levels VI or VII or facial lymph nodes | 2155 | 88.8 | 250 | 75.3 |  |
| Level VI lymph node(s) involved | 29 | 1.2 | 7 | 2.1 |  |
| Level VII lymph node(s) involved | 5 | 0.2 | 2 | 0.6 |  |
| Facial lymph node(s) involved | 1 | 0 | 1 | 0.3 |  |
| Level VI and VII lymph node(s) involved | 0 | 0 | 2 | 0.6 |  |
| Level VI and facial lymph node(s) involved | 2 | 0.1 | 0 | 0 |  |
| Level VII and facial lymph node(s) involved | 3 | 0.1 | 1 | 0.3 |  |
| Level VI, VII and facial lymph node(s) involved | 1 | 0 | 2 | 0.6 |  |
| Unknown | 230 | 9.5 | 67 | 20.2 |  |
| **Parapharyngeal, parotid, and suboccipital/retroauricular lymph nodes** |  |  |  |  | **<0.0001** |
| No involvement | 2049 | 84.5 | 234 | 70.5 |  |
| Parapharyngeal lymph node(s) involved | 101 | 4.2 | 14 | 4.2 |  |
| Parotid lymph node(s) _†_ involved | 22 | 0.9 | 8 | 2.4 |  |
| Suboccipital/retroauricular lymph node(s) involved | 2 | 0.1 | 2 | 0.6 |  |
| Parapharyngeal and parotid lymph node(s) involved | 6 | 0.2 | 1 | 0.3 |  |
| Parapharyngeal and suboccipital/retroauricular lymph node(s) involved | 2 | 0.1 | 0 | 0 |  |
| Parotid and suboccipital/retroauricular lymph node(s) involved | 2 | 0.1 | 1 | 0.3 |  |
| Parapharyngeal, parotid and suboccipital/retroauricular lymph node(s) involved | 2 | 0.1 | 1 | 0.3 |  |
| Unknown | 240 | 9.9 | 71 | 21.4 |  |

**Abbreviations:** Parotid lymph node(s) _†_, Parotid (preauricular, periparotid, and/or intraparotid) lymph node(s).
