# Supplementary material for: Pattern and prognosis of distant metastases in nasopharyngeal carcinoma: A large‐population retrospective analysis
Source: Cancer Med. 2020 Jul 10;9(17):6147–58. doi: 10.1002/cam4.3301 (PMC7476823; doi:10.1002/cam4.3301)
Supplement: Supplementary file 3 — Table S2‐S3 [file CAM4-9-6147-s003.docx]

**Supplementary table 2: Univariate Cox variance analysis of CSS and OS in NPC**

| **Variables** | **CCS** | | **OS** | |
| --- | --- | --- | --- | --- |
|  | **HR (95% CI**_†_**)** | **P value** | **HR (95% CI**_†_**)** | **P value** |
| **Age at diagnosis** |  | **<0.0001** |  | **<0.0001** |
| ≤50 | Reference |  | Reference |  |
| 50-70 | 1.663 (1.398-1.978) | <0.0001 | 1.748 (1.486-2.056) | <0.0001 |
| >70 | 3.052 (2.447-3.806) | <0.0001 | 3.413 (2.788-4.177) | <0.0001 |
| **Sex** |  | **<0.0001** |  | **<0.0001** |
| Male | Reference |  | Reference |  |
| Female | 0.709 (0.595-0.846) | <0.0001 | 0.712 (0.605-0.837) | <0.0001 |
| **Marital status** |  | **<0.0001** |  | **<0.0001** |
| Married | Reference |  | Reference |  |
| Unmarried | 1.328 (1.138-1.550) | <0.0001 | 1.356 (1.175-1.564) | <0.0001 |
| **Race recode** |  | **<0.0001** |  | **<0.0001** |
| White | Reference |  | Reference |  |
| Black | 1.097 (0.877-1.373) | 0.417 | 1.149 (0.937-1.408) | 0.181 |
| Other_‡_ | 0.671 (0.570-0.790) | <0.0001 | 0.666 (0.573-0.775) | <0.0001 |
| **Grade** |  | **<0.0001** |  | **<0.0001** |
| I | Reference |  | Reference |  |
| II | 1.128 (0.629-2.025) | 0.686 | 1.169 (0.679-2.011) | 0.574 |
| III | 0.696 (0.398-1.217) | 0.203 | 0.676 (0.402-1.139) | 0.141 |
| IV | 0.417 (0.237-0.736) | 0.003 | 0.439 (0.259-0.744) | 0.002 |
| **Histology** |  | **<0.0001** |  | **<0.0001** |
| KSCC | Reference |  | Reference |  |
| DNKSCC | 0.576 (0.479-0.693) | <0.0001 | 0.594 (0.501-0.705) | <0.0001 |
| UNKSCC | 0.383 (0.301-0.487) | <0.0001 | 0.415 (0.335-0.516) | <0.0001 |
| Other | 0.611 (0.494-0.756) | <0.0001 | 0.631 (0.518-0.768) | <0.0001 |
| **Stage group** |  | **<0.0001** |  | **<0.0001** |
| I | Reference |  | Reference |  |
| II | 1.039 (0.652-1.657) | 0.871 | 0.986 (0.662-1.468) | 0.945 |
| III | 1.904 (1.248-2.904) | 0.003 | 1.559 (1.085-2.241) | 0.016 |
| IV | 3.843 (2.567-5.754) | <0.0001 | 3.177 (2.254-4.478) | <0.0001 |
| **T stage** |  | **<0.0001** |  | **<0.0001** |
| T0 | Reference |  | Reference |  |
| T1 | 1.059 (0.263-4.276) | 0.935 | 0.859 (0.274-2.687) | 0.793 |
| T2 | 1.148 (0.283-4.663) | 0.846 | 0.920 (0.292-2.897) | 0.887 |
| T3 | 1.947 (0.483-7.852) | 0.349 | 1.479 (0.473-4.628) | 0.501 |
| T4 | 2.415 (0.600-9.713) | 0.214 | 1.881 (0.603-5.868) | 0.277 |
| TX | 2.912 (0.710-11.951) | 0.138 | 2.229 (0.699-7.105) | 0.175 |
| **N stage** |  | **<0.0001** |  | **<0.0001** |
| N0 | Reference |  | Reference |  |

**Supplementary table 2: Univariate Cox variance analysis of CSS and OS in NPC (continued)**

| N1 | 0.897 (0.721-1.115) | 0.328 | 0.940 (0.770-1.148) | 0.545 |
| --- | --- | --- | --- | --- |
| N2 | 1.047 (0.844-1.300) | 0.674 | 1.001 (0.818-1.225) | 0.992 |
| N3 | 1.524 (1.205-1.926) | <0.0001 | 1.516 (1.220-1.883) | <0.0001 |
| NX | 1.517 (0.891-2.585) | 0.125 | 1.827 (1.160-2.879) | 0.009 |
| **M stage** |  | **<0.0001** |  | **<0.0001** |
| M0 | Reference |  | Reference |  |
| M1 | 4.139 (3.497-4.899) | <0.0001 | 3.684 (3.140-4.322) | <0.0001 |
| **Scope Reg LN Sur_§_** |  | 0.723 |  | 0.595 |
| None | Reference |  | Reference |  |
| Reg LN biopsy | 1.026 (0.837-1.258) | 0.803 | 0.949 (0.783-1.151) | 0.595 |
| Reg LN removed | 0.904 (0.689-1.187) | 0.469 | 0.888 (0.691-1.141) | 0.351 |
| **Radiation therapy** |  | **<0.0001** |  | **<0.0001** |
| No | Reference |  | Reference |  |
| Yes | 0.254 (0.215-0.300) | <0.0001 | 0.257 (0.220-0.300) | <0.0001 |
| **Chemotherapy** |  | **<0.0001** |  | **<0.0001** |
| No | Reference |  | Reference |  |
| Yes | 0.565 (0.472-0.677) | <0.0001 | 0.553 (0.469-0.652) | <0.0001 |
| **Metastatic status** |  | **<0.0001** |  | **<0.0001** |
| No metastasis | Reference |  | Reference |  |
| Only bone metastasis | 3.540 (2.532-4.949) | <0.0001 | 3.310 (2.419-4.531) | <0.0001 |
| Only brain metastasis | 2.254 (0.724-7.014) | 0.161 | 2.489 (0.931-6.655) | 0.069 |
| Only liver metastasis | 6.082 (3.571-10.360) | <0.0001 | 5.328 (3.188-8.904) | <0.0001 |
| Only lung metastasis | 5.565 (3.718-8.330) | <0.0001 | 4.584 (3.068-6.848) | <0.0001 |
| Only DL metastasis | 2.316 (1.335-4.017) | 0.003 | 2.361 (1.437-3.880) | 0.001 |
| Dual-site metastasis | 4.543 (3.409-6.054) | <0.0001 | 3.971 (3.009-5.241) | <0.0001 |
| Tri-site metastasis | 5.300 (3.565-7.877) | <0.0001 | 4.710 (3.219-6.893) | <0.0001 |
| Tetra-site metastasis | 6.347 (2.834-14.211) | <0.0001 | 5.273 (2.357-11.795) | <0.0001 |
| Penta-site metastasis | 140.262 (19.041-1033.230) | <0.0001 | 106.943 (14.628-781.836) | <0.0001 |
| **Tumor size(mm)** |  | **<0.0001** |  | **<0.0001** |
| ≤30 | Reference |  | Reference |  |
| 30-60 | 1.745 (1.358-2.243) | <0.0001 | 1.753 (1.395-2.203) | <0.0001 |
| >60 | 3.432 (2.513-4.688) | <0.0001 | 3.279 (2.458-4.373) | <0.0001 |
| Microscopic focus_¶_ | 0.001 (0.000-2.044E+81) | 0.947 | 0.001 (0.000-1.159E+74) | 0.942 |
| **Size of Lymph Nodes(mm)** |  | **0.004** |  | **<0.0001** |
| No involved regional lymph nodes | Reference |  | Reference |  |
| ≤10 | 0.557 (0.321-0.966) | 0.037 | 0.594 (0.360-0.980) | 0.041 |
| 10-55 | 0.935 (0.763-1.147) | 0.522 | 0.971 (0.803-1.174) | 0.761 |
| >55 | 1.457 (1.077-1.973) | 0.015 | 1.593 (1.210-2.097) | 0.001 |

**Supplementary table 2: Univariate Cox variance analysis of CSS and OS in NPC (continued)**

| Microscopic focus_¶_ | 2.337 (0.327-16.719) | 0.398 | 2.079 (0.291-14.861) | 0.466 |
| --- | --- | --- | --- | --- |
| **Level I-III lymph nodes** |  | **0.003** |  | **0.005** |
| No involvement in levels I, II or III lymph nodes | Reference |  | Reference |  |
| Level I lymph node(s) involved | 1.179 (0.803-1.730) | 0.401 | 1.143 (0.798-1.637) | 0.466 |
| Level II lymph node(s) involved | 0.881 (0.715-1.084) | 0.232 | 0.935 (0.774-1.129) | 0.483 |
| Level III lymph node(s) involved | 1.525 (0.942-2.470) | 0.086 | 1.378 (0.864-2.199) | 0.178 |
| Levels I and II lymph node(s) involved | 1.228 (0.845-1.783) | 0.281 | 1.116 (0.779-1.599) | 0.549 |
| Level I and III lymph node(s) involved | 1.577 (0.586-4.244) | 0.367 | 1.699 (0.701-4.120) | 0.241 |
| Level II and III lymph node(s) involved | 1.135 (0.904-1.424) | 0.276 | 1.076 (0.869-1.332) | 0.502 |
| Level I, II and III lymph node(s) involved | 1.716 (1.253-2.352) | 0.001 | 1.724 (1.291-2.303) | <0.0001 |
| **Level IV-V and retropharyngeal lymph nodes** |  | **<0.0001** |  | **<0.0001** |
| No involvement in levels IV or V or retropharyngeal lymph nodes | Reference |  | Reference |  |
| Level IV lymph node(s) involved | 1.185 (0.838-1.675) | 0.337 | 1.205 (0.878-1.654) | 0.248 |
| Level V lymph node(s) involved | 1.196 (0.932-1.535) | 0.159 | 1.137 (0.901-1.436) | 0.280 |
| Retropharyngeal lymph node(s) involved | 0.915 (0.658-1.272) | 0.596 | 0.920 (0.679-1.246) | 0.590 |
| Level IV and V lymph node(s) involved | 2.014 (1.503-2.699) | <0.0001 | 1.878 (1.422-2.480) | <0.0001 |
| Level IV and retropharyngeal lymph node(s) involved | 1.352 (0.505-3.620) | 0.549 | 1.710 (0.764-3.827) | 0.192 |
| Level V and retropharyngeal lymph node(s) involved | 1.052 (0.617-1.793) | 0.853 | 1.017 (0.618-1.676) | 0.946 |
| Level IV, V and retropharyngeal lymph node(s) involved | 1.922 (1.226-3.015) | 0.004 | 1.814 (1.182-2.785) | 0.006 |
| **Level VI-VII and facial lymph nodes** | 1.101 (0.963-1.258) | 0.158 | 1.094 (0.965-1.241) | 0.161 |

**Supplementary table 2: Univariate Cox variance analysis of CSS and OS in NPC (continued)**

| **Parapharyngeal, parotid, and suboccipital/retroauricular lymph nodes** | 0.958 (0.814-1.127) | 0.605 | 1.020 (0.894-1.163) | 0.773 |
| --- | --- | --- | --- | --- |

**Abbreviations:** CI_†_, confidence interval; Other_‡_, American Indian, Alaska Native, Asian, Pacific Islander; Scope Reg LN Sur_§_, Scope regional lymph nodes surgery; Microscopic focus_¶_, Microscopic focus or foci only, no size of focus is given.

**Supplementary table 3: Multivariate Cox variance analysis of CSS and OS in NPC**

| **Variables** | **CCS** | | **OS** | |
| --- | --- | --- | --- | --- |
|  | **HR (95% CI**_†_**)** | **P value** | **HR (95% CI**_†_**)** | **P value** |
| **Age at diagnosis** |  | **<0.0001** |  | **<0.0001** |
| ≤50 | Reference |  | Reference |  |
| 50-70 | 1.631 (1.155-2.303) | 0.005 | 1.886 (1.366-2.605) | <0.0001 |
| >70 | 3.118 (2.057-4.726) | <0.0001 | 3.628 (2.463-5.344) | <0.0001 |
| **Grade** |  | **0.009** |  | **<0.0001** |
| I | Reference |  | Reference |  |
| II | 2.139 (0.845-5.415) | 0.109 | 2.096 (0.920-4.773) | 0.078 |
| III | 1.052 (0.440-2.515) | 0.909 | 0.959 (0.432-2.132) | 0.919 |
| IV | 1.144 (0.465-2.810) | 0.770 | 0.864 (0.384-1.945) | 0.725 |
| **Histology** |  | **0.039** |  | - |
| KSCC | Reference |  | - |  |
| DNKSCC | 0.730 (0.490-1.086) | 0.120 | - | - |
| UNKSCC | 0.500 (0.291-0.858) | 0.012 | - | - |
| Other | 0.956 (0.580-1.576) | 0.861 | - | - |
| **Stage group** |  | **<0.0001** |  | **<0.0001** |
| I | Reference |  | Reference |  |
| II | 1.903 (0.738-4.906) | 0.183 | 1.309 (0.618-2.775) | 0.482 |
| III | 4.010 (1.656-9.711) | 0.002 | 2.163 (1.070-4.371) | 0.032 |
| IV | 5.352 (2.225-12.874) | <0.0001 | 3.314 (1.657-6.629) | 0.001 |
| **Radiation therapy** |  | **<0.0001** |  | **<0.0001** |
| No | Reference |  | Reference |  |
| Yes | 0.350 (0.227-0.539) | <0.0001 | 0.333 (0.223-0.497) | <0.0001 |
| **Chemotherapy** |  | **0.011** |  | **0.044** |
| No | Reference |  | Reference |  |
| Yes | 0.586 (0.388-0.885) | 0.011 | 0.670 (0.454-0.990) | 0.044 |

**Supplementary table 3: Multivariate Cox variance analysis of CSS and OS in NPC (continued)**

| **Metastatic status** |  | **0.002** |  | **0.027** |
| --- | --- | --- | --- | --- |
| No metastasis | Reference |  | Reference |  |
| Only bone metastasis | 1.782 (0.858-3.704) | 0.122 | 1.496 (0.722-3.103) | 0.279 |
| Only brain metastasis | - | 0.948 | - | 0.960 |
| Only liver metastasis | 9.169 (3.154-26.653) | <0.0001 | 5.395 (2.068-17.035) | 0.001 |
| Only lung metastasis | 2.035 (0.724-5.723) | 0.178 | 1.787 (0.635-5.024) | 0.271 |
| Only DL metastasis | 0.752 (0.291-1.938) | 0.555 | 0.840 (0.355-1.987) | 0.691 |
| Dual-site metastasis | 2.779 (1.500-5.148) | 0.001 | 2.120 (1.186-3.788) | 0.011 |
| Tri-site metastasis | 1.662 (0.486-5.685) | 0.418 | 1.090 (0.360-3.299) | 0.878 |
| Tetra-site metastasis | 9.520 (1.168-77.564) | 0.035 | 11.804 (1.497-93.081) | 0.019 |
| **Tumor size(mm)** |  | **0.003** |  | **<0.0001** |
| ≤30 | Reference |  | Reference |  |
| 30-60 | 1.726 (1.198-2.487) | 0.003 | 1.855 (1.327-2.594) | <0.0001 |
| >60 | 1.998 (1.233-3.239) | 0.005 | 2.254 (1.446-3.514) | <0.0001 |
| **Level I-III lymph nodes** |  | - |  | 0.061 |
| No involvement in levels I, II or III lymph nodes | - |  | Reference |  |
| Level I lymph node(s) involved | - | - | 1.456 (0.653-3.248) | 0.358 |
| Level II lymph node(s) involved | - | - | 1.216 (0.867-1.705) | 0.258 |
| Level III lymph node(s) involved | - | - | 1.269 (0.548-2.939) | 0.578 |
| Levels I and II lymph node(s) involved | - | - | 1.290 (0.668-2.491) | 0.449 |
| Level I and III lymph node(s) involved | - | - | 2.284 (0.542-9.615) | 0.260 |
| Level II and III lymph node(s) involved | - | - | 0.934 (0.613-1.421) | 0.749 |
| Level I, II and III lymph node(s) involved | - | - | 2.429 (1.452-4.063) | 0.001 |

**Abbreviations:** CI_†_, confidence interval.
