# Supplementary material for: Pattern and prognosis of distant metastases in nasopharyngeal carcinoma: A large‐population retrospective analysis
Source: Cancer Med. 2020 Jul 10;9(17):6147–58. doi: 10.1002/cam4.3301 (PMC7476823; doi:10.1002/cam4.3301)
Supplement: Supplementary file 4 — Table S4 [file CAM4-9-6147-s004.docx]

**Supplementary table 4: Multivariate Cox variance analysis of CSS and OS in metastatic NPC with only one site**

| **Variables** | **CCS** | | **OS** | |
| --- | --- | --- | --- | --- |
|  | **HR (95% CI**_†_**)** | **P value** | **HR (95% CI**_†_**)** | **P value** |
| **Age at diagnosis** |  | 0.308 |  | 0.152 |
| ≤50 | Reference |  | Reference |  |
| 50-70 | 1.236 (0.706-2.163) | 0.459 | 1.210 (0.708-2.066) | 0.485 |
| >70 | 1.659 (0.868-3.169) | 0.126 | 1.813 (0.982-3.346) | 0.057 |
| **Sex** |  | 0.321 |  | 0.256 |
| Male | Reference |  | Reference |  |
| Female | 0.752 (0.428-1.320) | 0.321 | 0.731 (0.425-1.256) | 0.256 |
| **Histology** |  | **0.002** |  | **0.001** |
| KSCC | Reference |  | Reference |  |
| DNKSCC | 0.325 (0.176-0.600) | <0.001 | 0.342 (0.190-0.614) | <0.001 |
| UNKSCC | 0.449 (0.223-0.902) | 0.024 | 0.441 (0.224-0.870) | 0.018 |
| Other | 0.784 (0.447-1.377) | 0.397 | 0.771 (0.454-1.311) | 0.337 |
| **Radiation therapy** |  | 0.391 |  | 0.153 |
| No | Reference |  | Reference |  |
| Yes | 0.813 (0.506-1.306) | 0.391 | 0.725 (0.466-1.128) | 0.153 |
| **Chemotherapy** |  | **0.001** |  | **<0.001** |
| No | Reference |  | Reference |  |
| Yes | 0.405 (0.233-0.705) | 0.001 | 0.388 (0.231-0.651) | <0.001 |
| **Metastatic sites** |  | **0.043** |  | 0.083 |
| Only DL metastasis | Reference |  | Reference |  |
| Only bone metastasis | 2.227 (1.118-4.434) | 0.023 | 2.107 (1.126-3.943) | 0.020 |
| Only brain metastasis | 1.599 (0.412-6.200) | 0.498 | 1.808 (0.547-5.977) | 0.332 |
| Only liver metastasis | 3.142 (1.350-7.311) | 0.008 | 2.809 (1.273-6.198) | 0.011 |
| Only lung metastasis | 2.870 (1.386-5.942) | 0.005 | 2.332 (1.182-4.604) | 0.015 |

**Abbreviations:** CI_†_, confidence interval.
